# Fetal and maternal outcome after hyperimmunoglobulin administration for prevention of maternal–fetal transmission of cytomegalovirus during pregnancy: retrospective cohort analysis

**DOI:** 10.1007/s00404-020-05728-7

**Published:** 2020-08-04

**Authors:** Vera Seidel, Max Hackelöer, Rebecca C. Rancourt, Wolfgang Henrich, Jan-Peter Siedentopf

**Affiliations:** 1 Department of Obstetrics, Charité–Universitätsmedizin Berlin, Corporate Member of Freie Universität Berlin, Humboldt-Universität Zu Berlin, and Berlin Institute of Health, Augustenburger Platz 1, 13353 Berlin, Germany; 2 Division of ‘Experimental Obstetrics’, Clinic of Obstetrics, Charité–Universitätsmedizin Berlin, Corporate Member of Freie Universität Berlin, Humboldt-Universität Zu Berlin, and Berlin Institute of Health, Campus Virchow-Klinikum, Berlin, Germany

**Keywords:** Cytomegalovirus, Hyperimmunoglobulin, Pregnancy, Prevention of maternal, Fetal transmission

## Abstract

**Purpose:**

To determine the frequency of fetal infection as well as adverse pregnancy outcomes following antenatal hyperimmunoglobulin (HIG) treatment for primary cytomegalovirus (CMV) infection in pregnancy.

**Methods:**

In our observational cohort study, data from 46 women with a primary CMV infection during pregnancy were evaluated. Primary CMV infection was defined by seroconversion or the presence of CMV-IgM and low CMV-IgG avidity. All women received at least two or more infusions of HIG treatment (200 IU/kg). Congenital CMV infection (cCMV) was diagnosed by detection of CMV in amniotic fluid and/or neonatal urine. We compared the rate of maternal–fetal transmission from our cohort to data without treatment in the literature. The frequency of adverse pregnancy outcomes was compared to those of live-born infants delivered in our clinic.

**Results:**

We detected 11 intrauterine infections in our cohort, which correlates to a transmission rate of 23.9%. Compared to the transmission rate found in cases without treatment (39.9%), this is a significant reduction (*P* = 0.026). There were no adverse pregnancy outcomes in our cohort. The mean gestational age at delivery was 39 weeks gestation in treatment and control group.

**Conclusion:**

The administration of HIG for prevention of maternal–fetal CMV transmission during pregnancy seems safe and effective.

## Introduction

Congenital cytomegalovirus infection (cCMV) is the main cause of hearing loss and mental retardation in infants without genetic disorder [1]. The rate of cCMV differs depending on whether the infection of the fetus results from a recurrence of an earlier CMV infection of the mother or if the mother is primarily infected during pregnancy. In recurrent infections, the intrauterine transmission rate is estimated to be 0.5–1.2% [2–4]. After cCMV due to recurrent infection, newborns are rarely symptomatic [5, 6], although severe cases are reported [3]. In the cases of primary infection during pregnancy, the rate of cCMV is approximately 40% [7, 8]. The rate of transmission after primary infection in the first trimester in a German and Belgian historic cohort is estimated to be 35.2% [9]. This paper addresses only primary CMV infection in pregnancy. The prevalence of cCMV varies between 0.6 and 6.1% in developing countries [10] and 0.3% in Australia [11]. Since there is no general neonatal CMV screening in Germany, there is no available data on the prevalence for Germany. A retrospective data analysis in central Germany estimates the prevalence of clinically relevant Ccmv infection higher than 0.04% [12]. Approximately, 11% of congenitally infected infants have symptoms of cCMV [13]. The later the infection occurs in the mother during pregnancy, the higher the rate of transmission, but the lower the rate of symptomatic infants [14]. In a recent study of 138 children with cCMV, amniocentesis (AC) was performed in all pregnancies, predominantly at about 20–23 weeks gestation. In the cohort of infants with a negative CMV-DNA AC, thus with intrauterine infection later during the pregnancy, none of the children had long-term complications after birth. In contrast, in the cohort with a positive AC at about 20–23 weeks gestation, 14% suffered from long-term sequelae [15].

After fetal infection, in utero therapeutic options are limited [16]. In a non-randomized and non-controlled study, high-dose Valacyclovir was used for women with primary CMV infection during pregnancy which resulted in a better outcome for newborns with cCMV [17]. For in utero treatment with Valganciclovir, there are only case reports available [18].

For prevention of maternal–fetal transmission of CMV during pregnancy, hyperimmunoglobulin (HIG) treatment is controversially discussed and currently not generally recommended within international guidelines [19].

In Germany, since the publication of the guidelines on laboratory diagnostics of viral infections relevant in pregnancy [21], the number of voluntary CMV tests during pregnancy has increased. As a direct result, the consultation for positive tests in our outpatient clinic for infectious diseases in pregnancy rose accordingly [22]. In our outpatient clinic, we perform a thorough consultation for women with primary CMV infection during pregnancy, illustrating the limited and controversial data available. Women who requested an off-label HIG treatment for prevention of maternal–fetal transmission were offered a treatment in our clinic.

## Material and methods

### Patients and study design

All women who had received HIG between 01/2010 and 03/2017 in our outpatient clinic for infectious diseases in pregnancy at the Charité–Universitätsmedizin Berlin, Germany, a tertiary care hospital with the intention of prevention of maternal–fetal transmission, were contacted in January 2018 by mail. The study had local ethics committee (EA2/135/17) approval. Information was gathered from hospital charts/records, written questionnaires and partially telephone interviews. The women received information about the study purpose, an informed consent sheet for required signature and a data collection sheet about the pregnancy and infant’s outcome. Women were reminded about the study by phone and/or mail for a maximum of three times.

At the initial counselling, the option of HIG treatment was discussed with women with primary CMV infection during pregnancy. Primary infection of CMV was defined as either (a) confirmed seroconversion during pregnancy or (b) presence of CMV-IgM and low CMV-IgG avidity. CMV-IgGgB2 was registered if performed. Tests were inhomogeneous, as most women were referred from external gynecologists and presented their external laboratory values. In the cases of inconclusive data, the laboratory was contacted, or a control blood sample was taken in our center.

All women in the study collective received two or more infusions of HIG (200 IU/kg). HIG was given within a 2-week interval. After two infusions, avidity against CMV was tested. In the cases of increased avidity, no subsequent infusion was administered. For cases of similar avidity as was observed during the baseline measurements, a third infusion was administered. Each patient was informed about the possibility of AC to confirm or rule out intrauterine CMV infection. Congenital CMV infection was thus either diagnosed by detection of CMV in amniotic fluid and/or neonatal urine. According to our treatment protocol, all women had ultrasound follow-up during pregnancy.

Four women were lost to follow-up during pregnancy after AC was negative for CMV. One woman did not continue pregnancy after negative AC for other reasons. These five women were included in the transmission analysis, but not in the analysis on pregnancy outcome. Thus, our control group comprised of 82 women.

### Control group

For comparison of the rate of adverse pregnancy outcomes (i.e. pregnancy-induced hypertension, preeclampsia, eclampsia, preterm birth, AND low birth weight), we took a matched random sample from all births in our center. The Charité University Medical Center in Germany is a tertiary care hospital offering specialized treatment for any risk pregnancies, for children with heart diseases as well as mothers with infectious diseases and their children. For each viable birth within our clinic in the HIG-group, to create the control group, we matched and accessed the births that occurred before and afterwards. When a woman from our study group did not give birth in our clinic, we matched the two births from the same day before and after noon. We accessed the following parameters: pregnancy-induced hypertension, preeclampsia, eclampsia, completed weeks of gestation at birth, mode of birth, birth weight, head circumference at birth, length at birth, umbilical cord pH at birth, and other complications.

## Statistical analysis

The growth standard by Voigt et al., adjusting for gestational age at birth, was used to calculate age-adjusted and sex-adjusted *z* scores for weight, length, and head circumference at birth [23]. Results were given as mean or median [range or *n* (%)]. Groups were compared using the Fisher’s exact test with a significance level set at 0.05. Further testing was carried out by the Pearson Chi-square test for multiple division of outcomes. Statistical significance was set at *P* < 0.05.

The proportion of cases with transmission overall at any time during pregnancy was compared to the value of 39.9% (112/281) calculated by Feldman et al. [24]. Furthermore, transmission rates according to trimester of infection without HIG treatment for second trimester 42.0% (42/100) and third trimester 58.6% (17/29) were also taken from Feldman et al. The subgroup analysis of transmission before 20 weeks gestation and the start of HIG treatment before completed 15 weeks gestation was compared to the control group used by Kagan et al. [9] (35.2%) using the Fisher’s exact test with a significance level set at 0.05. Statistical analysis was performed using the software IBM® SPSS® Statistics Version 25.

## Results

### Study cohort

Between 01/2010 and 03/2017, a total of 62 women received HIG with the intention of prevention of maternal–fetal transmission in our outpatient clinic for infectious diseases. Most infections occurred in the first trimester (72%, 33 women). Mean interval between first suspicious lab test and HIG administration was 23 days. Mean gestational week at first HIG treatment was 17 weeks gestation. In the majority of cases, the HIG treatment was started within the second trimester, in nine cases within the first trimester and in three cases after 29 weeks gestation. Three of the women who received HIG were positive for IgG gB2. In those cases, the indication for HIG treatment was low avidity of IgG antibodies. HIG treatment was administered at least two times; however, six women received three doses. The third dosage was applied after 4 weeks in four cases and in two cases again after 2 weeks. Application was well tolerated, and no allergic reactions were reported. Data on maternal–fetal transmission were available for a total of 46 patients. Characteristics of the study population and their treatment course are summarized in Table 1.

**Table 1:** Characteristics and treatment course of study collective (*n* = 46)

Variable	Value
Maternal age (years) at delivery (*n* = 42^1^)	32 (21–44)
Gravida (*n* = 46)	2.3 (1–6)
Para (*n* = 46)	1.0 (0–4)
Pre-existing maternal diseases (*n* = 39)	Preexisting diabetes mellitus: 2 (5%) Neurodermatitis: 5 (13%) Arterial hypertension: 1 (3%) Autoimmune hepatitis: 1 (3%) Multiple sclerosis: 2 (5%) Hashimoto thyreoiditis: 1 (3%)
Indication for HIG treatment	
(a) Confirmed seroconversion during pregnancy	18 (39%)
(b) Presence of CMV-IgM and low CMV-IgG avidity	28 (61%)
Positive CMV-IgG gB2 before first HIG administration	3 (6.5%)
Completed weeks of gestation at first suspicious lab test (*n* = 46)	14 (5–31)
Completed weeks of gestation at first HIG administration (*n* = 46)	17 (6–34)
Days between first suspicious lab result and first HIG administration (*n* = 46)	23 (6–61)
First HIG administration within 21 days after first suspicious lab result	*N* = 26 (57%)
First HIG administration later than 21 days after first suspicious lab result	*N* = 20 (43%)
Number of HIG courses	
Two	40 (87.0%)
Three	6 (13.0%)
Number of AC performed	21 (45.7%)
Completed weeks of gestation at AC^2^ (*n* = 19)	22 (17–31)
Rate of maternal–fetal transmission (*n* = 46)	23.9% (11/46)
Detected by AC (*n* = 21)	9.5% (2/21)
Detected at delivery through neonates’ urine (*n* = 34)	29.4% (10/34)

Data are presented as median/mean (range) or *n* (%)

*HIG* hyperimmunoglobulin, *AC* amniocentesis

^1^Three women were lost to follow-up during the pregnancy, one woman decided for an induced abortion after positive AC

^2^For two women we know the result of the AC, but we do not know the gestational week the AC was performed

### Efficacy of transmission prophylaxis

In our cohort, 21 women decided for AC. Two ACs were positive for CMV in the amniotic fluid. Urine results after birth were only available for 34 neonates, of which ten were positive. In one case, a transmission occurred later in pregnancy after negative AC in 19 weeks gestation and the respective child was asymptomatic. The overall transmission rate was 23.9% (*P* = 0.026, in comparison to 39.9% in Israeli control group). When looking at transmissions before 23 weeks gestation confirmed via AC, the transmission rate is 7.1% with HIG treatment (*P* = 0.027, compared to 35.2% in the historic German Belgian control group). However, this subgroup is very small with only 14 ACs performed until 23 weeks gestation.

The transmission rate seems lower when HIG was administered within the first 21 days after the first suspicious lab result (19.2%, 5/26) compared to administration of HIG beyond 3 weeks (30.0%, 6/20), but this difference is not statistically significant (*P* = 0.307 in the Fisher’s exact test). The reduction in transmission of CMV from 39.9% in the Israeli cohort compared to 19.2% in our cohort remains significant in this subgroup analysis (*P* = 0.028). For an overview of transmission rates according to subgroup see Table 2.

**Table 2:** Efficacy of transmission prophylaxis with HIG

Trimester of maternal primary infection	Transmission rate without treatment % (*n*/*n*)	Transmission rate under HIG treatment	*P*
a. Overall transmission (detected by AC and/or urine of neonate)			
First trimester^1^ (*n* = 33)	35.2% (38/108)	24.2% (8/33)	0.169
Second trimester^2^ (*n* = 12)	42.0% (42/100)	16.7% (2/12)	0.079
Third trimester^2^ (*n* = 1)	58.6% (17/29)	100% (1/1)	0.600
Overall^2^ (*n* = 46)	39.9% (112/281)	23.9% (11/46)	0.026*
b. Transmission before 23 weeks gestation (detected by AC) under HIG treatment compared by study			
Kagan et al	35.2%^1^ (38/108)	2.5% (1/40)	< 0.000010*
Our cohort		7.1% (1/14)	0.027*
c. Overall transmission rate under HIG treatment when HIG was administered within 21 days after first suspicious lab result compared by study			
Kagan et al	39.9%^2^ (112/281)	7.5% (3/40)	< 0.000015*
Our cohort		19.2% (5/26)	0.028*

*AC* amniocentesis, *HIG* hyperimmunoglobulin

^1^Data from Kagan et al

^2^Data from Feldman et al

*P* values in one-sided Fisher’s exact test. Statistically significant (*P* ≤ 0.05) shown asterisked

Follow-up of the children was received for 39 children for a mean of 26 months (5–88 months). Of the 11 children born with cCMV, none had hearing impairments. Five children were treated postnatally for CMV. Only one child was symptomatic with microcephalus and development retardation.

### Rate of adverse pregnancy outcomes

No pregnancy-induced hypertension, preeclampsia, or eclampsia were observed in the study group or in the control group. There was one case of HELLP syndrome in the control group but none in the study group. Median completed gestational weeks at birth was 39 in both groups. There was no significant difference regarding neonates’ growth characteristics compared to our HIG treatment group, with rather a tendency towards higher absolute values in the HIG treatment group. A lower vaginal birth rate in the control group was observed which is probably due sampling method and hospital’s characteristic. See Table 3 for a detailed overview of pregnancy and obstetric outcomes.

**Table 3:** Comparison of pregnancy outcomes and obstetric complications between HIG-treatment group and control group

Characteristics	HIG treatment group (*N* = 41)	Control group (*N* = 82)	*P*
Median completed gestational weeks at birth (range)	39 (31–41)	39 (24–42)	0.082
GA at birth < 37 weeks (*n*) (%)	3 (7%)	13 (16%)	0.259
Mean BW in gram (range)	3484 (1695–4280)	3224 (775–4320)	0.674
Mean *z* score for BW (range)	0.31 (− 2.94–3.80)	0.04 (− 2.30–2.59)	0.469
Mean HC at birth in cm (range)	35.0 (30.0–37.0)	34.2 (24.0–38.0)	0.346
Mean *z* score for HC	0.14 (− 2.79–1.38)	− 0.13 (− 2.15–2.38)	0.290
Mean length at birth in cm (range)	51.4 (40.0–59.0)	50.3 (33.5–56.0)	0.152
Mean *z* score for length (range)	0.08 (− 3.96–3.09)	− 0.07 (− 1.78–2.29)	0.753
Mode of birth [*n* = 123]			0.003*
Vaginal	33 (80%)	37 (45%)	
Primary c-section	4 (10%)	25 (30%)	
Secondary c-section	4 (10%)	20 (24%)	
Mean umbilical cord pH (range) [*n* = 113]	7.27 (7.04–7.48)	7.26 (7.03–7.40)	0.439

*BW* birth weight, *HC* head circumference

*P*: *P* values in the two-sided Chi-square test according to Pearson. Statistically significant (*P* ≤ 0.05) shown asterisked

## Discussion

### Efficacy of HIG treatment for prevention of maternal–fetal transmission of CMV

The role of HIG for prevention of maternal–fetal transmission during primary CMV infection in pregnancy is controversially discussed [16, 19]. A non-randomized study by Nigro et al*.* reported a significant reduction of cCMV to 16% in comparison to 40% in the control group with no obstetric adverse events [8]. The subsequent randomized-controlled study by Revello et al*.* showed no significant effect of HIG (30% HIG group vs. 44% control group; *P* = 0.13) but raised concerns regarding a higher rate of obstetrical adverse events (preterm birth, preeclampsia and fetal growth retardation) in the HIG group compared to the placebo group (13% vs. 2%) [7]. Furthermore the study by Revello et al*.* could have reached significance level in a greater study collective according to van Leeuwen et al*.* [25]. The study by Revello et al*.* was criticized for various reasons [9]. First of all, a monthly HIG administration was performed although the half-life of HIG seems to be only 11 days [20]. Second, women with primary infection in the second trimester were treated and a median time interval between the diagnosis of infection and first HIG administration was five weeks. Third, the HIG dose administered was 100 IU per kg body weight. In a recent study by Kagan et al*.,* these controversial points were addressed and only women with primary infection during first trimester of their pregnancy were included. Treatment was initiated within 3 weeks, the gestational age at first HIG administration was ≤ 14 weeks and HIG was administered at 200 IU per kg body weight biweekly until 20 weeks gestation. Within this study protocol, the transmission rate was significantly lower compared to the rate in a historic cohort (7.5% intervention group vs. 35.2% in the control group; *P* < 0.0001) [9]. The transmission rate of 23.9% in our study was higher despite having the same HIG-dose as used by Kagan et al. In comparison to the Israeli control group with a transmission rate of 39.9% (112 transmission in 281 pregnancies with no HIG treatment), this reduction is significant (*P* = 0.026).

The risk for the neonate is highest if the infection occurs in the first trimester [26–30]. A recent study even suggests that only infections up to 23 weeks gestation lead to sequelae in the infant [15]. Hence, specifically the prevention of early transmission in pregnancy should be of high priority. When doing a subgroup analysis among the women who underwent AC within the first 23 weeks gestation, we also saw a lower rate of transmission (7.1%) than 35.2% in the historic German Belgian control group. The rate of 7.1% is nonetheless higher than 2.5% described by Kagan et al*.* First of all, our subgroup of only 14 ACs is very small. Second of all, this can possibly be due to less courses of treatment. Most of the women in our collective only received two courses of HIG, while Kagan et al. continued treatment until a median gestational age of 16.6 weeks and applied up to six courses of treatment [9]. Furthermore, treatment in our cohort was initiated delayed compared to Kagan et al. The median time lag between primary infection of the mother, approximated in this study by first suspicious lab result, and first HIG treatment course was 23 days. Only 57% of our patients received HIG within the first 3 weeks after CMV infection. This can be explained due to clinical reality and administrative barriers. Because serologic testing was performed in external outpatient clinics, referral of patients was delayed. Furthermore, sometimes in the cases of inconclusive blood results additional laboratory parameters were ordered from the laboratory until decision on HIG treatment was taken. After the decision for HIG due to administrative necessities such as the refunding of off-label treatment, a waiting period of at least three full working days had to be kept in order for the insurance company to check the indication.

### Safety of HIG treatment in pregnancy

The second controversially discussed aspect about HIG treatment during pregnancy are its potential side effects. Revello et al*.* reported obstetrical complications (preterm delivery, preeclampsia, and fetal growth restriction) in 7 of 53 women (13%) in the HIG group compared to 1 of 51 women (2%) in the placebo group (*P* = 0.06) [7]. Other authors found no increased rate of adverse events [9, 31, 32]. In our cohort, no preeclampsia occurred, and median completed gestational week was 39 weeks. Neonate growth parameters were not different in the HIG group compared to the control group. A lower c-section rate in the study group is most likely due to the sampling method where the time of birth was not known in the study group the control group was selected as the deliveries before and after noon in our center of tertiary care. This is typically the time for planned c-sections. Furthermore, the center of tertiary care is a referral center for women and children with illnesses necessitating c-sections as a mode of delivery. In conclusion, according to our data, no increased rate of adverse obstetric outcomes was detected and thus HIG application during pregnancy can be regarded as safe.

### Limitations of this study

The limitations in our study is the retrospective character and the lack of randomization. Furthermore, the cohort is very diverse. Time between infection and initiation of treatment is often later than advised by Kagan et al. Moreover, HIG was not only administered to first trimester infections. We suggest that a national or international prospective study with faster treatment initiation should follow.

### Strength of this study

We report results from the largest German cohort of HIG treatment during pregnancy, published so far. The results have implications for clinical management of CMV infection.

## Conclusion

The administration of HIG for prevention of maternal–fetal CMV transmission during pregnancy seems safe and effective. If HIG is applied for prevention of vertical CMV transmission, it should be administered bi-weekly in an adequate dosage until AC is performed. Treatment should be initiated as soon as possible after detection of CMV infection in pregnancy.

## References

[1] Ludwig A, Hengel H (2009). Epidemiological impact and disease burden of congenital cytomegalovirus infection in Europe. Euro Surveill.

[2] Fowler KB (1992). The outcome of congenital cytomegalovirus infection in relation to maternal antibody status. N Engl J Med.

[3] Henrich W (2002). Recurrent cytomegalovirus infection during pregnancy: ultrasonographic diagnosis and fetal outcome. Ultrasound Obstet Gynecol.

[4] Kagan KO, Hamprecht K (2017). Cytomegalovirus infection in pregnancy. Arch Gynecol Obstet.

[5] Wang C (2011). Attribution of congenital cytomegalovirus infection to primary versus non-primary maternal infection. Clin Infect Dis.

[6] Fowler KB, Stagno S, Pass RF (2003). Maternal immunity and prevention of congenital cytomegalovirus infection. JAMA.

[7] Revello MG (2014). A randomized trial of hyperimmune globulin to prevent congenital cytomegalovirus. N Engl J Med.

[8] Nigro G (2005). Passive immunization during pregnancy for congenital cytomegalovirus infection. N Engl J Med.

[9] Kagan KO (2018). Prevention of maternal-fetal transmission of CMV by hyperimmunoglobulin (HIG) administered after a primary maternal CMV infectionin early gestation. Ultrasound Obstet Gynecol.

[10] Lanzieri TM (2014). Systematic review of the birth prevalence of congenital cytomegalovirus infection in developing countries. Int J Infect Dis.

[11] Naing ZW (2016). Congenital cytomegalovirus infection in pregnancy: a review of prevalence, clinical features, diagnosis and prevention. Aust N Z J Obstet Gynaecol.

[12] Rutten H (2017). Congenital cytomegalovirus infection in Central Germany: an underestimated risk. Arch Gynecol Obstet.

[13] Kenneson A, Cannon MJ (2007). Review and meta-analysis of the epidemiology of congenital cytomegalovirus (CMV) infection. Rev Med Virol.

[14] Daiminger A, Bader U, Enders G (2005). Pre- and periconceptional primary cytomegalovirus infection: risk of vertical transmission and congenital disease. BJOG.

[15] Bilavsky E (2016). Clinical implications for children born with congenital cytomegalovirus infection following a negative amniocentesis. Clin Infect Dis.

[16] Buxmann H (2017). Primary human cytomegalovirus (HCMV) infection in pregnancy. Dtsch Arztebl Int.

[17] Leruez-Ville M (2016). In utero treatment of congenital cytomegalovirus infection with valacyclovir in a multicenter, open-label, phase II study. Am J Obstet Gynecol.

[18] Seidel V (2017). Intrauterine therapy of cytomegalovirus infection with valganciclovir: review of the literature. Med Microbiol Immunol.

[19] Rawlinson WD (2017). Congenital cytomegalovirus infection in pregnancy and the neonate: consensus recommendations for prevention, diagnosis, and therapy. Lancet Infect Dis.

[20] Hamprecht K, Kagan K-O, Goelz R (2014). Hyperimmune globulin to prevent congenital CMV infection. N Engl J Med.

[21] DVV and GfV (2014) Labordiagnostik schwangerschaftsrelevanter Virusinfektionen S2k-Leitlinie. http://www.awmf.org/uploads/tx_szleitlinien/093-001l_S2k_Labordiagnostik_schwangerschaftsrelevanter_Virusinfektionen_2014-05.pdf. 27 May 2016

[22] Seidel V (2017). Auswirkung der zunehmenden CMV-Serodiagnostik in der Schwangerschaft. Zeitschrift Geburtshilfe Neonatol.

[23] Voigt M (2006). Analysis of the neonatal collective in the federal republic of Germany 12th report: presentation of detailed percentiles for the body measurement of newborns. Geburtsh Frauenheilk.

[24] Feldman B (2011). Pregestational, periconceptional, and gestational primary maternal cytomegalovirus infection: prenatal diagnosis in 508 pregnancies. Am J Obstet Gynecol.

[25] van Leeuwen E, Oude Rengerink K, Pajkrt E (2014). Hyperimmune globulin to prevent congenital CMV infection. N Engl J Med.

[26] Adler SP (2012). Editorial commentary: primary maternal cytomegalovirus infection during pregnancy: do we have a treatment option?. Clin Infect Dis.

[27] Enders G (2011). Intrauterine transmission and clinical outcome of 248 pregnancies with primary cytomegalovirus infection in relation to gestational age. J Clin Virol.

[28] Lipitz S (2013). Risk of cytomegalovirus-associated sequelae in relation to time of infection and findings on prenatal imaging. Ultrasound Obstet Gynecol.

[29] Picone O (2013). A series of 238 cytomegalovirus primary infections during pregnancy: description and outcome. Prenat Diagn.

[30] Faure-Bardon V (2018). Sequelae of congenital cytomegalovirus following maternal primary infections are limited to those acquired in the first trimester of pregnancy. Clin Infect Dis.

[31] Nigro G (2015). Primary maternal cytomegalovirus infections during pregnancy: association of CMV hyperimmune globulin with gestational age at birth and birth weight. J Matern Fetal Neonatal Med.

[32] Chiaie LD (2018). No evidence of obstetrical adverse events after hyperimmune globulin application for primary cytomegalovirus infection in pregnancy: experience from a single centre. Arch Gynecol Obstet.

